# Evaluation of fetal diaphragm excursion and thickness in term pregnancies complicated with pre-gestational and gestational diabetes mellitus

**DOI:** 10.1186/s12978-022-01391-0

**Published:** 2022-04-02

**Authors:** Gokhan Acmaz, Fatma Ozdemir, Banu Acmaz, Yusuf Madendağ, Ilknur Çöl Madendag, Iptisam Ipek Muderris

**Affiliations:** 1 Department of Obstetrics and Gynecology, Erciyes University Faculty of Medicine, Kayseri, Turkey; 2 Department of Internal Medicine, Kayseri City Hospital, Kayseri, Turkey; 3 Department of Obstetrics and Gynecology, Kayseri City Hospital, Kayseri, Turkey

**Keywords:** Fetus, Pre-gestational diabetes mellitus, Gestational diabetes mellitus, Diaphragm, Ultrasound, Diabetes complications

## Abstract

**Background:**

Both pre-gestational (PGDM) and gestational diabetes mellitus (GDM) make pregnancy complicated. Moreover in the literature GDM and PGDM have been held responsible for respiratory morbidity in newborns. Diaphragm ultrasound (DUS) is a valuable and noninvasive method that provides an opportunity to examine the diaphragmatic morphology and function. This study examined the quality of fetal diaphragmatic contractions in pregnant women complicated with GDM and PGDM.

**Methods:**

A total of 105 volunteers who were separated into three groups; (1) A GDM group (n = 35), (2) a PGDM group (n = 35), and (3) a healthy non-diabetic control group (n = 35). All volunteers with the cephalic presentation and only male fetuses were examined in the 37th week of gestation. This cross sectional and case controlled study was performed at the perinatology clinic of the Erciyes University School of Medicine between 15.01.2020 and 01.08.2021. The thickness of fetal diaphragm (DT), diaphragmatic excursion (DE), diaphragm thickening fraction (DTF) and costodiaphragmatic angle (CDA) was measured and recorded by ultrasound and examined on the video frame during the inspiration and expiration phases of respiration.

**Results:**

Especially the PGDM group represented adversely affected diaphragm function parameters. DT inspiration, DT expiration, DE, CDA inspiration and DTF values were significantly different between PGDM and the control group. Neonatal intensive care unit (NICU) admission was high among babies who were born to pregnancies complicated with PGDM or GDM.

**Conclusions:**

The quality of fetal diaphragm movements is affected in pregnancies complicated with GDM and PGDM. The prolonged duration of diabetes may have additional adverse effects on diaphragm morphology and its function.

**Supplementary Information:**

The online version contains supplementary material available at 10.1186/s12978-022-01391-0.

## Plain language summary

The percentage of pre-gestational diabetes mellitus (PGDM) in pregnancy is 13–21% and the remaining part of diabetes is gestational diabetes mellitus (GDM). Both of the complications are related to respiratory problems at birth.

Until now, it was known that this situation was due to the lack of surfactant, which has a facilitating effect on the participation of the lungs in respiration. However, in this study, the diaphragm of the babies of patients with PGDM and GDM was examined. The thickness of fetal diaphragm, movements and function were evaluated via using ultrasound. As a result, it was determined that the diaphragm movements were impaired and the babies born from these patients needed more pediatric care.

This study will open horizon on new studies examining the functional capacity of the diaphragm in the future. In the future, it may be possible to decide which baby will need intensive care by examining the diaphragm.

**Supplementary Information:**

The online version contains supplementary material available at 10.1186/s12978-022-01391-0.

## Background

**Fig. 1:**
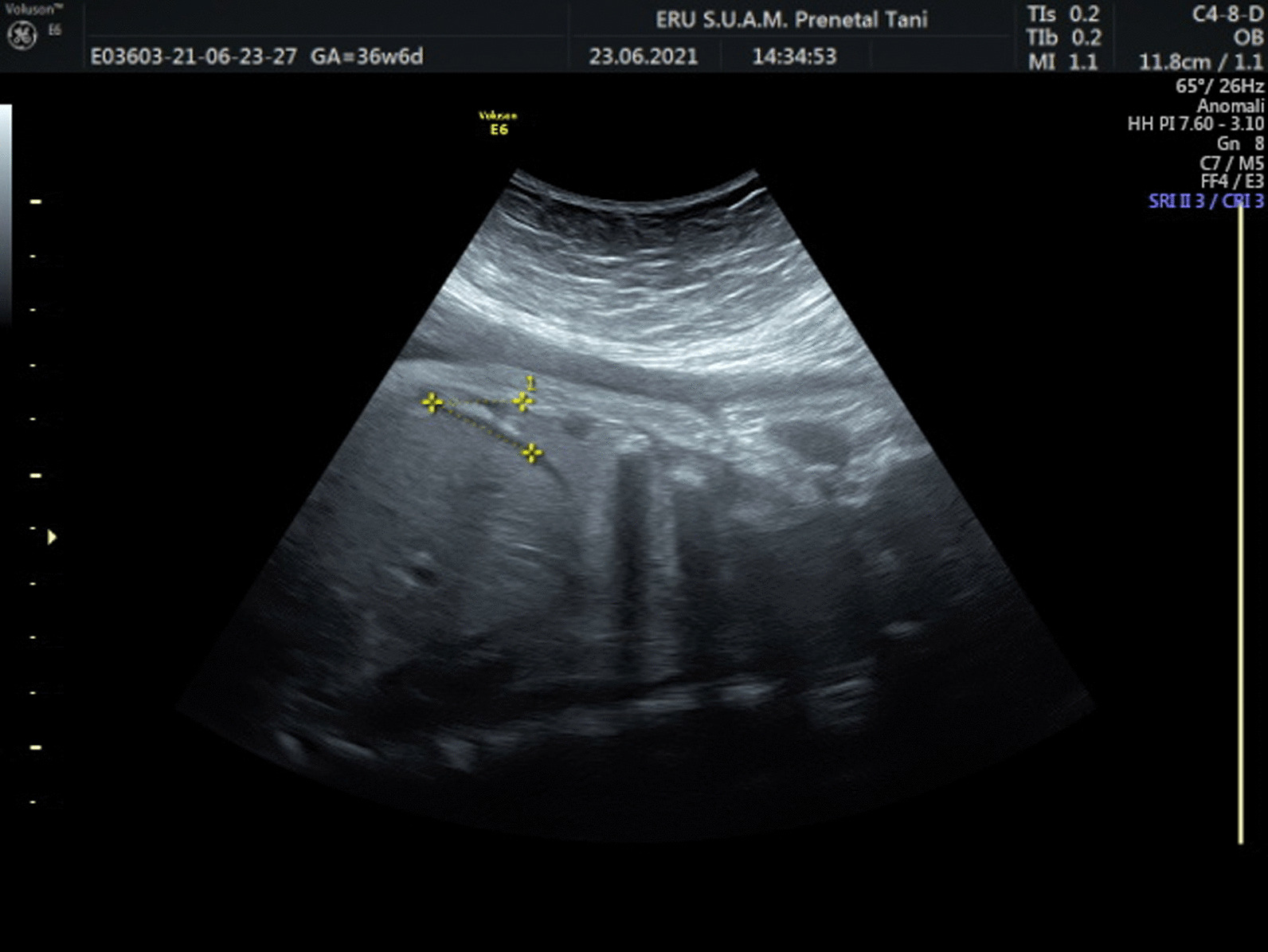
Exhibition of CDA

**Fig. 2:**
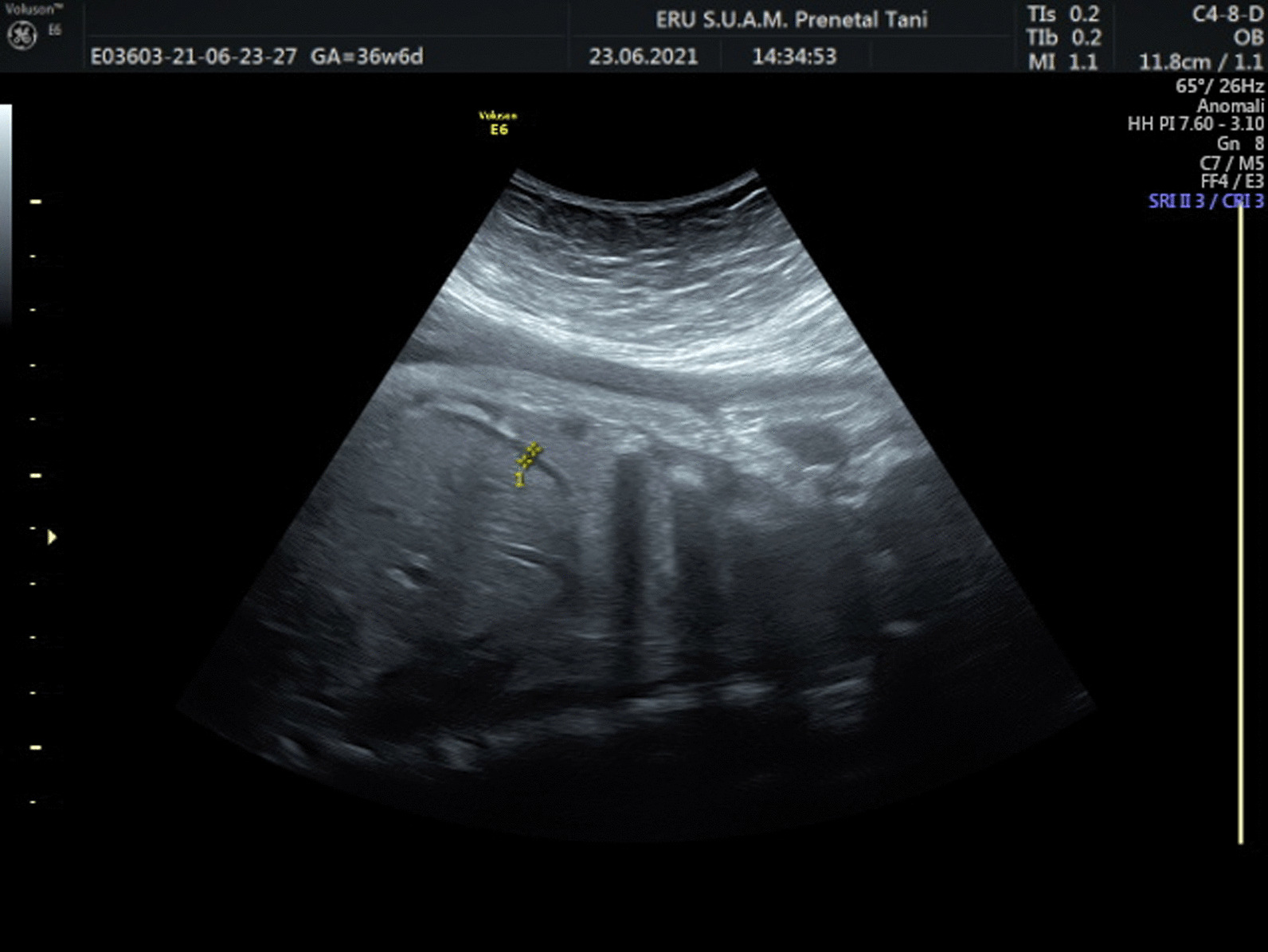
Exhibition of diaphragm thickness

Pre-Gestational (PGDM) and gestational diabetes mellitus (GDM), are two frequent medical conditions that complicate pregnancy due to high levels of blood glucose [1, 2]. There are well-known medical complications related to PGDM and GDM, including increased probability of cesarean, preterm labor, decreased levels of glucose (hypoglycemia), macrosomia, shoulder dystocia and fetal death [3]. Moreover both GDM and PGDM have been held responsible for respiratory morbidity in newborns [4–6]. Fetal hyperinsulinemia has been blamed for delayed pulmonary maturation [7] (Figs. 1, 2).

Diaphragm ultrasound (DUS) is a valuable and noninvasive method that provides an opportunity to examine the diaphragmatic morphology and function and has attracted the attention of researchers. Diaphragm excursion (DE), diaphragm thickness (DT), and costo-diaphragmatic angels (CDA) can be evaluated via using DUS. The diaphragmatic thickening ratio reflects the diaphragm’s contractile capacity that is related to its strength [8]. Moreover the efficiency of diaphragm contractions can be assessed via DT and diaphragm thickness fraction (DTF) measurements, obtained during the expiratory and inspiratory phases of respiration [9]. Authors have established that the DUS technique supplies valuable information for severe diaphragm weakness in critically ill adult patients [10, 11].

The effect of GDM or PGDM on fetal diaphragmatic function in pregnant patients is not clear in the literature. This study investigates the quality of fetal diaphragmatic contractions in pregnant women complicated with GDM and PGDM and compares these parameters with healthy pregnant volunteers.

## Methods

This study was performed at the perinatology clinic of the Erciyes University School of Medicine between 15.01.2020 and 01.08.2021 and classified as prospective, cross-sectional, and case-controlled. Signed written informed consent from all participants and Ethical Committee approval from the Erciyes University School of Medicine were obtained (no: 2019/652). This study has not been published elsewhere.

### Participants of the study

Patients who were admitted for the suspicion of pregnancy were evaluated. These patients were routinely screened for pregnancy tests. We evaluated 108 patients at the beginning who accepted to be a volunteer in the study. One of the volunteer in PGDM group were excluded because of diabetic ketoacidosis, one of the volunteer in control group were excluded because of data lost (she moved an other city) and one of the volunteer in GDM group declined to be participate. Remaining volunteers (n = 105) of this study were Caucasian origin and were followed up in our clinic after detecting heart beat up to delivery time. The gestational week of the study population was calculated using the last menstrual date. Gestational age was calculated according to first-trimester ultrasound reports in patients who did not remember the date of their last menstrual period. All volunteers were delivered via scheduled caesarean section at the 39th weeks of gestation with a cephalic presentation due to previous caesarean section.

According to the type of diabetes, participants were separated into three groups; (1) a GDM group (n = 35), (2) a PGDM group (n = 35), and (3) a healthy non-diabetic control group (n = 35). All GDM and healthy non-diabetic control group volunteers; were screened by 75 gr oral glucose tolerance test (OGTT) after 12 h fasting between the 24th and 28th weeks of gestation. The upper limits of fasting, first and second hour after 75 gr glucose administration were 92, 180 and 153 mg/dl, respectively [12]. Above this threshold, patients were diagnosed with GDM. All participants in the GDM group received diet and exercise therapy. Then they re-evaluated for glucose levels both fasting and post-prandial second hour. The upper limits of fasting and post-prandial second hour were 95 mg/dl and 120 mg/dl, respectively. We included only insulin-required participants in the GDM group. Patients in the PGDM group received examinations by an ophthalmologist and a nephrologist for retinopathy and nephropathy at the end of the second trimester (28th weeks of gestation) and 37th week of gestation. Moreover patients in the PGDM group were classified according to Sacks and Metzger’s definition and all volunteers in this group were Type 1 insulin-dependent DM without vascular complications [13].

### Exclusion criteria

All participants were examined in detail for fetal abnormalities and received toxoplasma, rubella, and cytomegalovirus tests at the 21th weeks of gestation. In the presence of any abnormality or positive test results, they were not accepted as suitable for the study. Because fetal sex is a confounder, only volunteers with male fetuses were included in the study. Patients with maternal fever, retinopathy, nephropathy, labor pain, non- cephalic presentation, female fetuses, preeclampsia/eclampsia, intrauterine growth retardation, oligohydroamnios, membrane rupture, chronic systemic diseases, chromosomal or fetal anomaly, twin gestation or more, placenta accereata, increata and percreata, intrahepatic cholestasis of pregnancy were excluded from the study. Volunteers who used steroids, narcotics, sedatives, tobacco, alcohol, or anti-psychotic were eliminated. Patients in the PGDM group received an examination by an ophthalmologist and a nephrologist for retinopathy and nephropathy at the end of the second trimester and 37th week of gestation. If diabetic volunteers were complicated with retinopathy and nephropathy, these patients were not included in the study. Additionally volunteers could not achieve normal glucose levels with insulin therapy were not included in the study in patients with PGDM and GDM.

Additional file 1: flowchart.

### Methods of diaphragmatic evaluation and DUSG timing

An obstetrician aimed to obtain horizontal views of the both left and right diaphragm and measure the angle of the costodiaphragmatic sinus during the examination. The diaphragm consists of two echogenic outer layers (pleura and peritoneum) and a non-echogenic middle layer (central layer). Video records of diaphragmatic examinations, both inspiration and expiration states were obtained from all volunteers by an obstetrician (FO) who was blinded to the diagnosis of the volunteers. Then measurements were performed by reviewing records frame by frame. We obtained the thickness of the fetal diaphragm (DT) at two time points of the respiratory cycle (end-inspiration thickness and end-expiration thickness of the fetal diaphragm). Both right and left diaphragm has different movement abilities due to the position of the liver; thus, all examinations were performed bilaterally and measurements were illustrated as mean values of both sides. In the presence of gasping or ‘picket-fence’ breathing of fetus FO did not evaluate that respiratory cycle. All volunteers with the cephalic presentation were examined in the 37th week of gestation after ruling out nephropathy and retinopathy.

### Measurement of other diaphragm parameters

During the expiration and inspiration phases of the respiratory cycle, the diaphragm moves at the highest and lowest points in the fetal chest. The average distances between the highest and lowest points of the diaphragm on two respiratory cycles were recorded and calculated. The distance between these two points illustrates the ability of diaphragmatic movement, and it is called diaphragmatic excursion (DE). We evaluated another diaphragmatic function marker, ‘diaphragm thickening fraction’ (DTF), using a formula (end-inspiration thickness- end-expiration thickness/end-expiration thickness × 100) [14]. Then the costodiaphragmatic angle (CDA) was measured on the same video frame during the inspiration and expiration phases of respiration.

### Other parameters

None of the volunteers had fetal distress, and all volunteers underwent planned caesarean section at the 39th week of gestation with general anesthesia due to a previous uterine scar. For determining fetal lactate, pH, oxygen, and carbon dioxide, (pO_2_, pCO_2_, SO_2_) levels, arterial cord blood was obtained after the fetus’s expulsion. Apgar scores, fetal hypoxia, hypotonia, transient tachypnea, mild respiratory distress syndrome, NICU requirement, and other factors were evaluated by a pediatrician. An author (Çİ) collected required demographic and clinical data before USG examination.

### Determining sample size and statistical analysis

For calculating sample size, means, standard deviations, and reference values were taken from the article “Adverse fetal outcomes in patients with intra-uterine-growth-retardation (IUGR) are related with fetal diaphragm evaluation parameters” [14]. We found 29 volunteers necessary when we assumed that power = 0.80 and alpha = 0.05. Because of possible data loss and dropouts, 36 volunteers were included in the study. We excluded one volunteer from each group.

To test the normality assumption of the data, the Shapiro–Wilk test was used. Variance homogeneity assumption was tested with the Levene test. Values were expressed as mean ± standard deviation, median (25th percentile–75th percentile), or n (%). One-Way ANOVA, Chi Square, and Kruskal–Wallis H tests were performed to compare differences between groups. Tukey, and Mann–Whitney U tests were used for the multiple comparisons. p < 0.05 probability value was considered as statistically significant. All calculations were made using PASW Statistics 18 software.

## Results

Of the 105 pregnant women enrolled in the study, 35 were in the GDM group, 35 were in the pre-gestational DM group, and 35 were in the healthy control group. Table 1 provides the demographic characteristics among groups.

**Table 1:** Demographic characteristics of PGDM, GDM, and control groups

	GDM group (n = 35)	Pre-gestational DM (n = 35)	Control group (n = 35)	p-value
Maternal age (year)	33.3 ± 6.4^a^	34.9 ± 5.3^a^	29.0 ± 5.1^b^	< 0.001
Gravity	3 (2–4)	3 (2–4)	2 (2–4)	0.509
Parity	1 (1–2)	2 (1–2)	1 (1–2)	0.506
Abortion	0 (0–1)	0 (0–1)	0 (0–1)	0.956
Mean gestational age at ultrasound evaluation (week)	37 (37–37.1)	37 (37–37.2)	37 (37–37.2)	0.834

*Different superscripts indicate statistically significant difference

Both PGDM and GDM volunteers were older than the control group. Other parameters are homogeneously distributed. Table 2 provides the comparison of fetal diaphragm measurement parameters among groups.

**Table 2:** Comparisons of fetal diaphragm functional parameters among groups

	GDM group (n = 35)	Pre-gestational DM (n = 35)	Control group (n = 35)	p-value
DT inspiration (mm)	2.61 ± 0.17^ab^	2.69 ± 0.17^a^	2.53 ± 0.18^b^	0.001
DT expiration (mm)	2.45 ± 0.18^ab^	2.54 ± 0.15^a^	2.35 ± 0.20^b^	< 0.001
DE (mm)	5.55 (5.30–5.80)^a^	5.20 (5.10–5.45)^b^	5.90 (5.60–6.20)^c^	< 0.001
CDA Inspiration (degrees)	61.71 ± 3.78^a^	57.86 ± 3.42^b^	62.09 ± 3.79^a^	< 0.001
CDA expiration (degrees)	50 (46–51)	51 (46–53)	50 (48–52)	0.662
DTF	6.53 (5.56–8.57)^ab^	5.84 (5–7.14)^a^	6.67 (5.88–9.43)^b^	0.045

*Different superscripts indicate statistically significant difference

The PGDM group primarily represented adversely affected diaphragm measurement parameters. DT inspiration, DT expiration, DE, CDA inspiration, and DTF values significantly differed between PGDM and the control group.

Table 3 provides the comparison of delivery outcomes among groups.

**Table 3:** Comparisons of delivery outcomes among groups

	GDM group (n = 35)	Pre-gestational DM (n = 35)	Control group (n = 35)	p-value
Fetal weight (g)	3280 (3110–3610)	3370 (2940–3870)	3370 (3050–3520)	0.909
Umblical artery Ph	7.34 ± 0.052	7.33 ± 0.058	7.34 ± 0.055	0.636
Base excess (mmol/lt)	− 1.20 (− 1.90–0.80)	− 0.50 (− 1.20–0.80)	− 0.50 (− 1.20–0.50)	0.302
PO_2_ (mmHg)	91 (90–94)^a^	90 (89–92)^ab^	90 (87–91)^b^	0.018
PCO_2_ (mmHg)	45(38.90–47)^a^	47 (45–50)^b^	48 (45–50)^b^	0.007
SO_2_ (%)	95(92.10–96)	95 (93–96,30)	95 (93–97)	0.959
Lactate (mmol/dl)	0.90 (0.70–1.42)	0.80 (0.60–1.10)	0.80 (0.60–1.10)	0.516
1 min Apgar score	8 (8–8)	8 (8–8)	8 (8–8)	0.368
5 min Apgar score	10 (10–10)	10 (10–10)	10 (10–10)	0.368
NICU admission (n%)	4 (%11)^a^	6 (%17)^a^	0 (%0)^b^	0.045

*Different superscripts indicate statistically significant difference

Babies who were born to both PGDM and GDM volunteers, showed significantly high NICU admission.

## Discussion

In the presence of GDM and pre-gestational DM, diaphragm USG might be helpful to understand the quality of breathing efforts in newborns. Good quality diaphragm movements are an important sub-type of diaphragm movements.

### Results of previous studies about DUSG, PGDM, GDM and their babies

DUSG has been used as a marker of well being in the intensive care unit (ICU) patients moreover it is purposive to determine suitable patients for liberation from mechanic ventilator [15, 16]. DE and DTF, which are DUSG components, exhibit breathing effort and active muscular contractions respectively [16].

Previously published studies illustrated and discussed the structure of the diaphragm. Authors showed that lateral regions of the right and left diaphragm do not participate entirely in the movement; however, medial and middle regions play a critical role [17]. Sonographic measurements showed that CDA at the end of expiration state was not different among groups; however, CDA during inspiration state was significantly affected in the PGDM group. This situation may be related to ineffective diaphragmatic contractions. Moreover the expiration state of respiratory cycles occurs passively within the relaxation of diaphragm muscles [18]. Thus we believe that CDA at the inspiration state of the respiratory cycle is a better indicator of diaphragmatic contractions. Other study findings supported this situation, and we found DE and DTF parameters were significantly affected in the PGDM group, which showed movement ability and contraction capacity, respectively [9, 14].

### Clinical significance

Four fetuses (% 11) in the GDM group, six fetuses (% 17) in the PGDM group were admitted to NICU. However, none of the fetuses in the control group required NICU admission. Fetal weight, gestational age and umbilical artery pH values were not different among the groups but naturally both PGDM and GDM group volunteers were older than control group. Because gestational age was not different among groups, we thought NICU admission or other parameters was not affected from maternal age.

The present study found that DT inspiration, DT expiration, and DTF were significantly higher and DE and CDA inspiration were significantly lower in the PGDM group than in the control group. In addition umbilical artery blood gas PO_2_, umbilical artery blood gas PCO_2_, and NICU admission rates were statistically different among groups. In the literature, GDM and pre-gestational DM have been accepted as risk factors for neonatal respiratory morbidity [4, 5, 19]. Glucose imbalance and fetal hyperinsulinemia have been held responsible for delayed pulmonary maturation [20]. Moreover, these complications are presumably related to insufficient surfactant synthesis, due to maternal hyperglycemia [21, 22]. In the literature, authors examined the effect of insulin on surfactant protein A (SP-A) and surfactant protein B (SP-B) secretion. They found that insulin lowers both surfactant protein (protein A and protein B) productions in lung tissue [23, 24]. Additionally, reduced fluid clearance in the fetal lungs, augmented by increased cesarean delivery rates was another problem [25–27]. As far as we know, this is the first study that shows GDM and PGDM have adverse effects on fetal diaphragm thickness and diaphragm function in term pregnancies. In this case, we can argue that diabetes harms diaphragm movements and diaphragm structure.

## Conclusions

Our results indicated that surfactant synthesis and quality of fetal diaphragm movements are affected in pregnancies complicated with GDM and pre-gestational DM. Thus, prolonged duration of diabetes may have an extra adverse effect on diaphragm morphology and its function. We can suggest that cephalic presentation, male gender, small sample size can be accepted study limitations.

## Supplementary Information

**Additional file 1. **Flowchart.

## Data Availability

The data used to support the findings of this study are available on request from the corresponding author.

## Abbreviations

PGDM: Pre-gestational diabetes mellitus
GDM: Gestational diabetes mellitus
DUS: Diaphragm ultrasound
DT: Thickness of fetal diaphragm
DE: Diaphragmatic excursion
DTF: Diaphragm thickening fraction
CDA: Costodiaphragmatic angle
IUGR: Intrauterine growth retardation
NICU: Neonatal intensive care unit
FO: Fatma Ozdemir
OGTT: Oral glucose tolerance test

## References

[1] American Diabetes Association (2015). Classification and diagnosis of diabetes. Diabetes Care.

[2] Sahin E, Col Madendag I, Sahin ME, Madendag Y, Acmaz G, Muderris II (2019). Effect of vitamin D deficiency on the 75 g oral glucose tolerance test screening and insulin resistance. Gynecol Endocrinol.

[3] Metzger BE, Lowe LP, Dyer AR, Trimble ER, Chaovarindr U, Coustan DR, Hadden DR, McCance DR, Hod M, McIntyre HD, Oats JJ, Persson B, Rogers MS, Sacks DA, HAPO Study Cooperative Research Group (2008). Hyperglycemia and adverse pregnancy outcomes. N Engl J Med.

[4] Bental Y, Reichman B, Shiff Y, Weisbrod M, Boyko V, Lerner-Geva L, Mimouni FB, Collaboration With the Israel Neonatal Network (2011). Impact of maternal diabetes mellitus on mortality and morbidity of preterm infants (24–33 weeks’ gestation). Pediatrics.

[5] Becquet O, El Khabbaz F, Alberti C, Mohamed D, Blachier A, Biran V, Sibony O, Baud O (2015). Insulin treatment of maternal diabetes mellitus and respiratory outcome in late-preterm and term singletons. BMJ Open.

[6] Billionnet C, Mitanchez D, Weill A, Nizard J, Alla F, Hartemann A, Jacqueminet S (2017). Gestational diabetes and adverse perinatal outcomes from 716,152 births in France in 2012. Diabetologia.

[7] McGillick EV, Morrison JL, McMillen IC, Orgeig S (2014). Intrafetal glucose infusion alters glucocorticoid signalling and reduces surfactant protein mRNA expression in the lung of the late gestation sheep fetus. Am J Physiol Regul Integr Comp Physiol.

[8] Randerath WJ, Young P, Boentert M, Spießhöfer J, Herkenrath SD (2019). Evaluation of respiratory muscle strength by diaphragm ultrasound: normative values, theoretical considerations and practical recommendations. Pneumologie.

[9] McCool FD, Conomos P, Benditt JO, Cohn D, Sherman CB, HoppinJr FG (1997). Maximal inspiratory pressures and dimensions of the diaphragm. Am J RespirCrit Care Med.

[10] Lerolle N, Guérot E, Dimassi S, Zegdi R, Faisy C, Fagon JY, Diehl JL (2009). Ultrasonographic diagnostic criterion for severe diaphragmatic dysfunction after cardiac surgery. Chest.

[11] Mariani LF, Bedel J, Gros A, Lerolle N, Milojevic K, Laurent V, Hilly J, Troché G, Bedos JP, Planquette B (2016). Ultrasonography for screening and follow-up of diaphragmatic dysfunction in the ICU: a pilot study. J Intensive Care Med.

[12] Metzger BE, Gabbe SG, Persson B, Buchanan TA, Catalano PA, Damm P, Dyer AR, Leiva Ad, Hod M, Kitzmiler JL, Lowe LP, McIntyre HD, Oats JJ, Omori Y, Schmidt MI, International Association of Diabetes and Pregnancy Study Groups Consensus Panel (2010). International association of diabetes and pregnancy study groups recommendations on the diagnosis and classification of hyperglycemia in pregnancy. Diabetes Care.

[13] Sacks DA, Metzger BE (2013). Classification of diabetes in pregnancy: time to reassess the alphabet. Obstet Gynecol.

[14] Acmaz G, Ozdemir F, Sahin E, Sahin ME, Madendag Y, Demir TB, Karakas E, Muderris II, Nisari M, Bayraktar E (2021). Adverse fetal outcomes in patients with IUGR are related with fetal diaphragm evaluation parameters. Paediatr Respir Rev.

[15] Matamis D, Soilemezi E, Tsagourias M, Akoumianaki E, Dimassi S, Boroli F, Richard JC, Brochard L (2013). Sonographic evaluation of the diaphragm in critically ill patients. Technique and clinical applications. Intensive Care Med.

[16] Goligher EC, Fan E, Herridge MS, Murray A, Vorona S, Brace D, Rittayamai N, Lanys A, Tomlinson G, Singh JM, Bolz SS, Rubenfeld GD, Kavanagh BP, Brochard LJ, Ferguson ND (2015). Evolution of diaphragm thickness during mechanical ventilation impact of inspiratory effort. Am J Respir Crit Care Med.

[17] de la Quintana FB, Alcorta BN, Pérez MF (2017). Ultrasound evaluation of diaphragm function and its application in critical patients, mechanical ventilation and brachial plexus block. Revista Española de Anestesiología y Reanimación (English Edition).

[18] Kokatnur L, Vashisht R, Rudrappa M. DiaphragmDisorders. 2020 Aug 8. In: StatPearls [Internet]. Treasure Island (FL): StatPearls Publishing; 2021.

[19] Kawakita T, Bowers K, Hazrati S, Zhang C, Grewal J, Chen Z, Sun L, Grantz KL (2017). Increased neonatal respiratory morbidity associated with gestational and pregestational diabetes: a retrospective study. Am J Perinatol.

[20] McGillick EV, Morrison JL, McMillen IC, Orgeig S (2014). Intrafetal glucose infusion alters glucocorticoid signaling and reduces surfactant protein mRNA expression in the lung of the late-gestation sheep fetus. Am J Physiol Regul Integr Comp Physiol.

[21] Gewolb IH, O'Brien J (1997). Surfactant secretion by type II pneumocytes is inhibited by high glucose concentrations. Exp Lung Res.

[22] Gewolb IH (1996). Effect of high glucose on fetal lung maturation at different times in gestation. Exp Lung Res.

[23] Miakotina OL, Goss KL, Snyder JM (2002). Insulin utilizes the PI 3-kinase pathway to inhibit SP-A gene expression in lung epithelial cells. Respir Res.

[24] Miakotina OL, Dekowski SA, Snyder JM (1998). Insulin inhibits surfactant protein A and B gene expression in the H441 cell line. Biochim Biophys Acta.

[25] Pinter E, Peyman JA, Snow K, Jamieson JD, Warshaw JB (1991). Effects of maternal diabetes on fetal rat lung ion transport. Contribution of alveolar and bronchiolar epithelial cells to Na+, K(+)-ATPase expression. J Clin Invest.

[26] Robert MF, Neff RK, Hubbell JP, Taeusch HW, Avery ME (1976). Association between maternal diabetes and the respiratory-distress syndrome in the newborn. N Engl J Med.

[27] Hibbard JU, Wilkins I, Sun L, Gregory K, Haberman S, Hoffman M, Kominiarek MA, Reddy U, Bailit J, Branch DW, Burkman R, Gonzalez Quintero VH, Hatjis CG, Landy H, Ramirez M, VanVeldhuisen P, Troendle J, Zhang J, Consortium on Safe Labor (2010). Respiratory morbidity in late preterm births. JAMA.

