# Supplementary material for: Evaluation of fetal diaphragm excursion and thickness in term pregnancies complicated with pre-gestational and gestational diabetes mellitus
Source: Reprod Health. 2022 Apr 2;19:87. doi: 10.1186/s12978-022-01391-0 (PMC8977043; doi:10.1186/s12978-022-01391-0)
Supplement: Supplementary file 1 — Additional file 1. Flowchart. [file 12978_2022_1391_MOESM1_ESM.docx]

**Evaluation of Fetal Diaphragm Excursion and Thickness in Term Pregnancies Complicated with Pre-gestational and Gestational Diabetes Mellitus**

Assessed for eligibility (n= 108)

Excluded (n= 3)

♦  Not meeting inclusion criteria (n= 1)

♦  Declined to participate (n= 1 )

♦  Other (n= 1)

## Enrollment

## Allocation

Randomized (n= 105 )

Allocated to intervention A^[[1]](#footnote-1)^ (n=36 )

Analysed (n= )
♦ Excluded from analysis (give reasons) (n= )

Analysed (n= )
♦ Excluded from analysis (give reasons) (n= )

Analysed (n= )
♦ Excluded from analysis (give reasons) (n= )

Analysed (n= )
♦ Excluded from analysis (give reasons) (n= )

Analysed (n= )
♦ Excluded from analysis (give reasons) (n= )

Analysed (n= )
♦ Excluded from analysis (give reasons) (n= )

## Analysis

♦ Received allocated intervention (n=36 )

♦ Did not receive allocated intervention (give reasons) (n= 0 )

Allocated to intervention A^[[2]](#footnote-2)^ (n=36 )

Analysed (n= )
♦ Excluded from analysis (give reasons) (n= )

Analysed (n= )
♦ Excluded from analysis (give reasons) (n= )

Analysed (n= )
♦ Excluded from analysis (give reasons) (n= )

Analysed (n= )
♦ Excluded from analysis (give reasons) (n= )

Analysed (n= )
♦ Excluded from analysis (give reasons) (n= )

Analysed (n= )
♦ Excluded from analysis (give reasons) (n= )

## Analysis

♦ Received allocated intervention (n=36 )

♦ Did not receive allocated intervention (give reasons) (n= 0 )

Allocated to intervention A^[[3]](#footnote-3)^ (n=36 )

Analysed (n= )
♦ Excluded from analysis (give reasons) (n= )

Analysed (n= )
♦ Excluded from analysis (give reasons) (n= )

Analysed (n= )
♦ Excluded from analysis (give reasons) (n= )

Analysed (n= )
♦ Excluded from analysis (give reasons) (n= )

Analysed (n= )
♦ Excluded from analysis (give reasons) (n= )

Analysed (n= )
♦ Excluded from analysis (give reasons) (n= )

## Analysis

♦ Received allocated intervention (n=36 )

♦ Did not receive allocated intervention (give reasons) (n= 0 )

GDM GROUP PGDM GROUP CONTROL GROUP

**Female:** Allocated to intervention (n=36 )

♦ Received allocated intervention (n=36 )

♦ Did not receive allocated intervention (give reasons) (n=0 )

**Female:** Allocated to intervention (n=36 )

♦ Received allocated intervention (n= 36 )

♦ Did not receive allocated intervention (give reasons) (n= 0 )

**Female:** Allocated to intervention (n= 36 )

♦ Received allocated intervention (n=36 )

♦ Did not receive allocated intervention (give reasons) (n= 0 )

Lost to follow-up (give reasons) (n= 0)

Discontinued intervention (give reasons) (n=1 )

Lost to follow-up (give reasons) (n= 0 )

Discontinued intervention (give reasons) (n= 1 )

## Follow-Up

Analysed (n=35 )
♦ Excluded from analysis (give reasons) (n=0 )

Analysed (n= 35 )
♦ Excluded from analysis (give reasons) (n= 0)

## Analysis

Female: One of the volunteers changed her mind. She declared that she did not want to take part in the study.

**Female:** She excluded from the study due to diabetic ketoacidosis

**Female:** Analysed (n=35 )
♦ Excluded from analysis (give reasons) (n= 0 )

**Female:** Analysed (n= 35)
♦ Excluded from analysis (give reasons) (n= 0 )

Lost to follow-up (give reasons) (n= 1 )

Discontinued intervention (give reasons) (n= 0 )

**Female:** She moved to Ankara

Analysed (n=35 )
♦ Excluded from analysis (give reasons) (n=0 )

**Female:** Analysed (n=35 )
♦ Excluded from analysis (give reasons) (n= 0 )

1. [↑](#footnote-ref-1)
2. [↑](#footnote-ref-2)
3. [↑](#footnote-ref-3)
